# Can opportunities be enhanced for vaccinating children in home visiting programs? A population-based cohort study

**DOI:** 10.1186/s12889-015-1926-8

**Published:** 2015-07-07

**Authors:** Michael R Isaac, Mariette Chartier, Marni Brownell, Dan Chateau, Nathan C Nickel, Patricia Martens, Alan Katz, Joykrishna Sarkar, Milton Hu, Elaine Burland, ChunYan Goh, Carole Taylor, PATHS Equity Team Members

**Affiliations:** Department of Community Health Sciences, University of Manitoba, Winnipeg, Manitoba Canada; Manitoba Centre for Health Policy, University of Manitoba, Winnipeg, Manitoba Canada

**Keywords:** Vaccination programs/utilization, Pediatric, Child, Humans, Home visit, Propensity score, Early childhood development programs

## Abstract

**Background:**

Home visiting programs focused on improving early childhood environments are commonplace in North America. A goal of many of these programs is to improve the overall health of children, including promotion of age appropriate vaccination. In this study, population-based data are used to examine the effect of a home visiting program on vaccination rates in children.

**Methods:**

Home visiting program data from Manitoba, Canada were linked to several databases, including a provincial vaccination registry to examine vaccination rates in a cohort of children born between 2003 and 2009. Propensity score weights were used to balance potential confounders between a group of children enrolled in the program (n = 4,562) and those who were eligible but not enrolled (n = 5,184). Complete and partial vaccination rates for one and two year old children were compared between groups, including stratification into area-level income quintiles.

**Results:**

Complete vaccination rates from birth to age 1 and 2 were higher for those enrolled in the Families First program [Average Treatment Effect Risk Ratio (ATE RR) 1.06 (95 % CI 1.03–1.08) and 1.10 (95 % CI 1.05–1.15) respectively]. No significant differences were found between groups having at least one vaccination at age 1 or 2 [ATE RR 1.01 (95 % CI 1.00–1.02) and 1.00 (95 % CI 1.00–1.01) respectively). The interaction between program and income quintiles was not statistically significant suggesting that the program effect did not differ by income quintile.

**Conclusions:**

Home visiting programs have the potential to increase vaccination rates for children enrolled, despite limited program content directed towards this end. Evidence-based program enhancements have the potential to increase these rates further, however more research is needed to inform policy makers of optimal approaches in this regard, especially with respect to cost-effectiveness.

## Background

Vaccinations are one of the greatest public health achievements of the 20th century [1]. However, vaccine preventable disease outbreaks continue to occur and highlight the importance of high vaccination coverage rates and herd immunity. One subset of the population that has been shown to have lower vaccination coverage rates are children living in poverty, putting them at higher risk for acquiring a vaccine preventable infection [2–5]. A promising intervention to increase coverage rates in low socio-economic populations is a home visiting program that includes home vaccination or vaccine promotion with direct referral to a vaccination provider [6, 7]. Public Health organizations have strongly recommended these interventions alone or as part of larger community based programs to increase vaccination rates, with a recent systematic review reporting a median increase in vaccination rates of 10 percentage points [6]. However, these programs can be resource-intensive and costly when applied only to improve vaccination coverage, compromising the uptake of this intervention overall [7].

Much more common in North America are home visiting programs focused on improving outcomes related to cognition, socio-emotional development and maltreatment in children. Evidence for these programs has been well documented on a range of health outcomes [8–11]. Typically, a pre-defined curriculum is carried out with parents over several visits to achieve improvements in both child and parent outcomes [8]. Programs are varied in their content and goals, but many aim to improve child health, including vaccination rates. Despite home visits with parents over a span of several months to several years, many home visiting programs do not employ home vaccination or direct referral for vaccination despite strong recommendations to do so and clear benefits [4]. Instead, many programs choose to take a more passive approach, limiting content to the provision of well-child health information, such as age appropriate vaccination schedules. Questions remain as to the effect of this approach on vaccination coverage rates for those enrolled in such programs.

The literature examining this area is mixed. Kendrick et al. conducted a systematic review and meta-analysis of home visiting programs and their effect on vaccination rates and concluded that such programs were not effective at improving vaccination rates (OR 1.17, 95 % CI 0.33–4.17) [4]. However, the home visiting interventions included in the analysis were heterogeneous and the extent to which vaccination content was included, or not, in each program was unclear. Other studies have found a positive effect of home visiting on vaccination rates. Koniak-Griffin et al. reported a higher percentage of vaccination for one-year-olds in an early intervention program compared to a group with traditional public health nursing care (96 % vs. 86 %) [12]. This study did not account for potential confounders and the sample size was small (n = 97). El-Mohandes et al. completed a randomized controlled trial of a home visiting program and assessed outcomes including preventive pediatric health care services and found a significant effect on up-to-date vaccinations at 9 months (OR 2.2, 95 % CI 1.09–4.53) with an apparent dose–response effect as those who had more than 30 visits with study personnel had a higher likelihood of up-to-date vaccination (OR 3.63, 95 % CI 1.58–8.33) [13]. This trial was also small, including 286 families, and supplemented home visits with hospital-based group sessions, limiting the external validity.

To address the uncertainty in the literature, we conducted a population-based cohort study to examine the effect of a home visiting program on pediatric vaccination rates and assessed if this relationship differed by socio-economic status. The Families First home visiting program examined in this study provides home visits with the intent of supporting healthy child development, improving parent–child relationships, connecting families with their communities and decreasing child maltreatment [14]. In addition to a curriculum addressing these topics with parents, information about vaccine schedules is provided and parents are reminded of the need for childhood vaccinations. (See Appendix) The number of children in Manitoba having a full series of vaccinations is variable, however recent data have approximated rates of complete vaccination for age 1 and 2 to be 78 % and 60 % respectively [15].

This study aims to fill a gap in the literature by analyzing a large and representative sample of at-risk children (n = 9,745). It also aims to comprehensively address potential confounders by linking multiple databases with Families First home visiting data, thereby providing a large number of covariates to use in the statistical analysis. Programs aimed at improving uptake of interventions can result in overall improvements but also increase inequities [16]; therefore, this analysis also examined equity in vaccination rates, which to our knowledge has not been previously reported for those in home visiting programs.

## Methods

### The program

The Families First home program was initiated in Manitoba, Canada, in 1999. It was modeled on Hawaii’s Healthy Start program and uses the Growing Great Kids Inc. curriculum [17, 18]. Approximately 80 % of all births in the province of Manitoba are screened for eligibility for the program, initially with a brief newborn screen of biological, social, and demographic risk factors and then, for those screened at-risk, followed up with a comprehensive risk factor assessment using a parent survey. This parent survey is based on the Kempe Family Stress Inventory that covers a variety of domains, including psychiatric history, criminal and substance abuse history, childhood history of care, emotional functioning, attitudes towards and perception of child, discipline of child, and level of stress in the parent’s life [19]. Parents’ responses on this survey are used to calculate a risk score; herein referred to as the ‘parent survey score.’ Families with a parent survey score ≥ 25 are eligible for the program, which involves working with a trained paraprofessional home visitor overseen by a public health nurse. The average length of time in the program is 18 months and the average number of visits per month is just over two, although this varies depending on the family’s needs.

### Data source

This study was undertaken in Manitoba, a province of approximately 1.2 million people geographically located near the centre of Canada. Residents of Manitoba receive health care services under a universal program of insurance coverage funded by the provincial government, including a program of publicly funded vaccinations for all Manitobans. Data for this study were derived from the PATHS (Pathways to Health and Social Equity) Data Resource, a unique database developed for the PATHS program of research containing anonymized individual-level data from the Population Health Research Data Repository, which is housed at the Manitoba Centre for Health Policy (MCHP) [20]. The PATHS Data Resource includes data provided by Manitoba Health, Healthy Living and Seniors; Manitoba Jobs and the Economy; Manitoba Family Services; Healthy Child Manitoba; the Winnipeg Regional Health Authority; Statistics Canada; and Manitoba Housing and Community Development. The data were not ‘openly available’, but rather permission for its use was requested and received from each of the individual data providers, as well as the Health Information Privacy Commission (HIPC) of the Government of Manitoba. However, other researchers interested in using these data are encouraged to apply for access; more information on applying for access can be found, elsewhere [20].

The PATHS Data Resource comprises population-based individual-level data on the health and social services use for over 99 % of all Manitoba children. The validity of the data included in the PATHS resource has been well documented [21–23]. Individual-level data from several databases across multiple domains were linked using encrypted identifying numbers. Databases used in the analyses were:

1. The Manitoba Immunization Monitoring System (MIMS), which is an electronic vaccination registry that captures pediatric and adult vaccinations through a physician billing system for publicly funded vaccinations, as well as vaccinations entered into the system by other health care providers;
2. The Manitoba Health Insurance Registry, which includes demographic information on all residents registered for health care;
3. Employment and Income assistance data;
4. Families First Screen (including the newborn screen and home visiting data);
5. Small Area-level Census data (dissemination area) on employment, education, income and lone-parent status combined in an index of socio-economic status [24], and used to create income quintiles of the population.

### Population

This study included children born between January 1, 2003 and December 31, 2009 who had at least three risk factors on the newborn screen, program data, and an actual or imputed parent survey score ≥ 25. Families First program data were collected by public health nurses and the Healthy Child Manitoba Office and included the newborn screen, parent survey, and program implementation data.

**Fig. 1:**
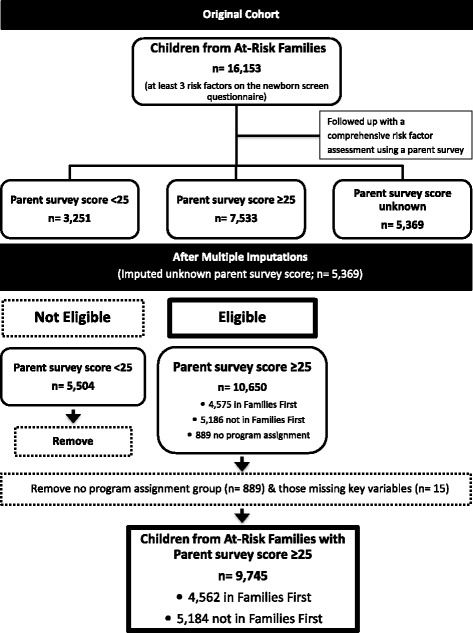
Flow diagram showing study participant selection

Figure 1 outlines the process of selecting study participants. The original Families First database had information on 16,153 families. There was no parent survey score for 5,369 families. These families’ parent survey scores were estimated using multiple imputation (explained below) because excluding them could bias the results of our study. In practice, the parent survey may not have been done due to public health nurse challenges (e.g., large workloads, lack of experience with at-risk families) or family characteristics such as lack of trust of service providers, no address, no telephone, or addictions issues (personal communication-Marion Ross). Imputing the missing parent survey scores added 2,736 families to the comparison group, 104 to the program group, and 2,253 were excluded because the imputed scores were lower than 25. An additional 904 families were excluded because the program assignment variable and other key variables confirming program entry were missing. The final sample included 4,562 children from families in the program (the intervention group) and 5,184 children from families who were eligible for the program, but did not receive it (the comparison group). Based on statistics kept by Healthy Child Manitoba, we estimate that of the 5,186 eligible families (imputed or documented parent survey score ≥25) that did not participate in the program, 37 % of families refused the program, 11 % did not enter because the program was full and another 52 % were never offered the program because the parent survey questionnaire was unable to be completed for reasons discussed above.

### Variables

#### Dependent variables

The outcomes studied were complete vaccinations and at least one vaccination for one- and two-year-old children. The criteria for complete vaccination from birth to age 1 and 2 years can be found in Table 1. At least one vaccination encompasses all of those who had a least one vaccination for each immunogen, based on the criteria in Table 1.

**Table 1:** Definition of complete vaccination from birth to age 1 and 2 years old

Age at vaccination	Type of vaccines							
	Diphtheria	Pertussis	Tetanus	Polio	HiB^a^	Pneumococcus	MMR^b^	Varicella
Complete vaccination from birth to age 1 prior to October 1, 2004								
2 months	x	x	x	x	x			
4 months	x	x	x	x	x			
6 months	x	x	x	(x)	x			
Complete vaccination from birth to age 1 after October 1, 2004								
2 months	x	x	x	x	x	x		
4 months	x	x	x	x	x	x		
6 months	x	x	x	(x)	x	x		
Complete vaccination from birth to age 2 prior to October 1, 2004								
2 months	x	x	x	x	x			
4 months	x	x	x	x	x			
6 months	x	x	x	(x)	x			
12 months							x	
18 months	x	x	x	x	x			
Complete vaccination from birth to age 2 after October 1, 2004								
2 months	x	x	x	x	x	x		
4 months	x	x	x	x	x	x		
6 months	x	x	x	(x)	x	x		
12 months							x	x
18 months	x	x	x	x	x	x		

^a^Haemophilus Influenzae type b

^b^Measles, Mumps, Rubella

(x) This dose is not needed routinely, but can be given

#### Independent variables

Table 2 presents the list of confounding variables included in these analyses. These variables were selected due to their potential influence on both exposure (i.e., participation in the Families First program) and outcome (vaccination). The degree to which families in the program differ from families in the comparison group, on both measured and unmeasured characteristics, may bias estimates of the program effect on vaccination. Adjusting for the variables listed in Table 2 serves to create comparable exposed/unexposed groups, based on observed characteristics.

**Table 2:** Description of independent variables

Variables and data source	Description
a. Variables from the Families First newborn screen	
Prenatal screening	Whether the parent was screened prenatally for the program as opposed to after the birth of the child
No prenatal care before 6 months	Mother did not attend prenatal care before 6 months gestation
Alcohol and/or drug use	Any alcohol and/or drug use by the mother during pregnancy
Maternal Substance abuse	Current substance abuse by mother
Smoking during pregnancy	Maternal smoking during pregnancy
Social Isolation	Lack of social support and/or isolation related to culture, language or geography
Maternal Low education	Mother’s highest level of education completed being less than grade 12
Single parent family	Parent or guardian not currently in common-law relationship or married
Social Assistance	Family on social assistance/income support or having significant financial difficulties
Relationship distress	Parent reporting relationship distress
Schizophrenia (Mother)	Mother has schizophrenia or bipolar affective disorder
Depression and/or anxiety (Mother)	Mother has depression (including postpartum) and/or anxiety disorder
Antisocial (Father)	Father has antisocial behavior
Antisocial (Mother)	Mother has antisocial behavior
Mental disability (Mother)	Mother has mental disability
Family History of disability	Family history of a disability not detectable at birth that could affect development (eg. deafness, mentally disabled/challenged)
Violence between parents	Current or history of violence between parenting partners
Child abuse mom	Mother having a history of child abuse or neglect
b. Variables from Families First home visiting data	
Parent survey scores	Cumulative score of items on parent survey
Average duration of enrollment	Average duration of enrollment in the program based on program discharge data (in months)
Average Number of home visits	Average number of home visits per month based on program discharge data
c. Other Variables	
SEFI-2	Socioeconomic factor index – version 2. An index based on Canadian census data that reflects non-medical social determinants of health.
Mother’s age at first birth	Calculated from the population registry database using mother’s date of birth and date of first birth.

### Missing data

A considerable amount of the Families First program data were missing (38.8 %) due to parents declining to provide certain information, staff not able to obtain information, coding error and lost records. We used multiple imputation to account for missing data to ensure our cohort was representative of at-risk children in the population.

Multiple imputation can provide a valid statistical technique for handling missing data, which accounts for the inherent uncertainty caused by missing values [25, 26]. Multiple imputation uses known data from included covariates to estimate values for covariates with missing data. We used data from the covariates listed in Table 2 in the multiple imputation. Markov Chain Monte Carlo methods were used to fill in missing values 10 times, generating 10 complete data sets using the SAS procedure MI. We analyzed each of the 10 complete datasets using the analytic methods described below. We combined the results from the 10 complete datasets using the SAS procedure MIANALYZE. Analyzing 10 multiple imputed data sets and then combining the results using MIANALYZE (a) reflects the fact that uncertainty remains with respect to the unknown values and (b) provides statistically valid inferential results [25, 26].

### Statistical testing

There were 4 steps to the analyses.

1. Estimating each child’s propensity score for being enrolled in the Families First Program;
2. Testing the program’s impact on (a) complete vaccination at one year and two years of age and (b) at least one vaccination at one year and two years of age, after adjusting for confounding through application of propensity scores;
3. Stratifying results by income quintiles and testing for effectiveness by income quintile by adding an interaction term to the models; and
4. Calculating Gamma values to estimate findings’ sensitivity to hidden confounding [27].

Logistic regression was used to estimate propensity scores for all children eligible for the program (n = 9,745). The propensity score is the child’s predicted probability of receiving the Families First program given his/her observed characteristics.

Three treatment effects were of interest: the average treatment effect (ATE), the average treatment effect on the treated (ATT), and the average treatment effect on the untreated (ATU). The ATE is the effect of the Families First program on vaccination among the entire eligible population. The ATT is the average effect of the program on vaccination among those who actually received the Families First intervention. The ATU is the anticipated effect of the program on vaccination if the comparison group had instead received the Families First program. These concepts are explained in significant detail, elsewhere [28, 29].

The propensity scores were used to create three sets of inverse-probability-of-treatment weights (IPTWs), each set corresponding to the three treatment effects of interest as described above. IPTWs were applied to the models to balance differences between the intervention and comparison groups’ potential confounders; this is one method for adjusting for measured confounding [28].

Standardized differences were calculated prior to and after the weighting procedure for both the Families First group and those who were eligible but not enrolled in the Families First program to assess the effect of the weighting procedure between groups. Standardized differences measure effect sizes between two groups and are independent of the sample size, which makes them valuable in comparing baseline covariates for studies that use propensity scoring [30]. A standardized difference of less than 10 % is generally accepted as being satisfactory with respect to homogeneity between intervention and control groups for a given covariate [31].

Generalized linear modeling was used to estimate the predicted probability for each vaccination outcome of interest, by exposure-status. Separate models were estimated for each outcome and were used to estimate risk ratios and risk differences. Multiplicative models were used to estimate risk ratios and additive models were used to estimate risk differences [32]. Measures of precision (e.g., 95 % confidence intervals and standard errors) came from the models and an a priori significance level of p < 0.05 was used. All analyses were performed using SAS® version 9.2 [33].

Finally, we determined whether the effectiveness of the program differed by income quintile by introducing an interaction term (urban income quintile x Families First program) to the models. We examined the interaction term using income quintile as a continuous variable and only considered families living in urban areas for this analysis. The urban income quintile variable was made up of five values, 1 for the lowest income quintile and 5 for the highest. Rural areas were not included because they tend to have more mixing of income groups compared to urban groups, which have more homogenous income groups within a given geographical boundary [34].

With propensity score methods, it is assumed that estimates are not sensitive to unmeasured confounding. We conducted a sensitivity analysis to test this assumption. A gamma value was generated, indicating the strength of unmeasured confounding required to invalidate statistically significant results. In other words, Gamma is the hidden confounding which would make the relationship between Families First and vaccination appear significant when, in fact, it is not significant [27, 28]. A gamma value was calculated for each statistically significant result.

## Results

Table 3 outlines the distribution of the variables for those enrolled and those not enrolled in the Families First program. After the weighting procedure is applied, the percentage of risk factors is similar across children from families enrolled and the comparison group. Table 4 also shows that the weighting procedure was successful, as standardized differences for covariates in the weighted groups do not exceed 10 %.

**Table 3:** Characteristics of families enrolled (Intervention group) versus families eligible for program but not enrolled (Comparison group) prior-to and after weighting^a^ (n = 4562 in Families First program; n = 5184 not in Families First program)

	Un-weighted		Average treatment effect (ATE)	
	In Families First	Not in Families First	In Families First	Not in Families First
Alcohol and/or drug use (%)	37.96	38.80	38.32	38.31
Antisocial father (%)	4.98	4.22	4.77	4.76
Antisocial mother (%)	2.19	1.80	1.95	1.93
Child abuse mom (%)	26.40	18.61	22.20	22.30
Depression and/or anxiety mother (%)	36.23	31.57	34.20	34.06
Family history of disability (%)	5.34	4.69	4.98	4.94
Social isolation (%)	16.50	9.46	12.81	12.89
Maternal low education (%)	52.18	53.17	53.13	52.95
Mental disability mother (%)	1.69	1.00	1.34	1.43
No prenatal care before 6 months (%)	6.78	9.73	8.38	8.36
Prenatal screening (%)	17.49	7.72	12.28	12.29
Relationship distress (%)	26.00	19.03	22.51	22.51
Schizophrenia mother (%)	1.65	1.55	1.65	1.64
Single parent family (%)	42.55	47.73	45.64	45.36
Smoking during pregnancy (%)	49.79	56.01	52.86	52.86
Social assistance (%)	66.44	69.07	67.65	67.69
Maternal substance abuse (%)	3.30	2.73	3.00	2.95
Violence between parents (%)	9.50	8.13	8.72	8.82
Mother’s age at first birth (mean)	21.09	20.75	20.94	20.94
Parent survey scores (mean)	38.45	36.66	37.38	37.35
Socioeconomic factor index II (mean)	0.53	0.73	0.63	0.63

^a^Weighting refers to the use of inverse-probability-of-treatment weights to adjust for potential confounders

**Table 4:** Standardized differences^a^ between families enrolled (Intervention group) versus families eligible for program but not enrolled (Comparison group) prior-to and after weighting^b^ (n = 4562 in Families First program; n = 5184 not in Families First program)

	Unweighted (%)	Average treatment effect (ATE) (%)	Average treatment effect for treated group (ATT) (%)	Average treatment effect for untreated group (ATU) (%)
Mother’s age at first birth	7.41	0.09	1.51	1.47
Alcohol and/or drug use	1.72	0.04	0.43	0.36
Antisocial father	3.64	0.04	1.73	1.70
Antisocial mother	2.75	0.14	0.74	0.43
Child abuse mom	18.74	0.23	0.33	0.16
Depression and/or anxiety mother	9.87	0.29	1.42	1.86
Family history of disability	2.95	0.18	0.60	0.21
Social isolation	21.05	0.24	0.63	0.21
Maternal low education	1.98	0.36	1.01	1.57
Mental disability mother	5.93	0.79	1.65	0.21
No prenatal care before 6 months	10.74	0.08	0.17	0.25
Parent survey scores	16.11	0.26	2.65	2.08
Relationship distress	16.74	0.07	0.94	0.92
Schizophrenia mother	0.80	0.14	0.61	0.82
Prenatal screening	29.74	0.06	0.16	0.22
Socioeconomic factor index II	20.68	0.67	0.73	1.85
Single parent family	10.42	0.57	0.16	1.16
Smoking during pregnancy	12.50	0.09	1.10	1.00
Social assistance	5.64	0.11	0.75	0.84
Maternal substances abuse	3.32	0.25	0.50	0.14
Violence between parents	4.86	0.32	0.28	0.36

^a^Standardized difference is the difference in measured effect sizes between the two groups

^b^ Weighting refers to the use of inverse-probability-of-treatment weights to adjust for potential confounders

Vaccination outcomes from birth to age one and two can be seen in Table 5. There were significant differences between the Families First group and the comparison group when assessing complete vaccination from birth to age one [ATE RR 1.06, ATT RR 1.05, ATU RR 1.06 (95 % CI 1.03–1.08)]. There were no significant differences between the groups for having at least one vaccination from birth to age one (ATE, ATT, ATU RR 1.01 (95 % CI 1.00–1.02)].

**Table 5:** Predicted probability, risk ratios and risk differences between families enrolled and not enrolled in the Families First program, 2003–2009 (n = 4562 in Families First program; and n = 5184 eligible but not in Families First program)

	Predicted probability		Risk Difference	95 % CI	Risk Ratio	95 % CI
	In FF	Not in FF				
Complete vaccination for 1-year old						
Unweighted	0.78	0.72	0.06^a^	0.04–0.07	1.08^a^	1.05–1.10
ATE: Average Treatment Effect	0.77	0.73	0.04^a^	0.02–0.06	1.06^a^	1.03–1.08
ATT: Average Treatment Effect for Treated Group	0.78	0.74	0.04^a^	0.02–0.06	1.05^a^	1.03–1.08
ATU: Average Treatment Effect for Untreated Group	0.77	0.72	0.04^a^	0.02–0.06	1.06^a^	1.03–1.08
At Least One Vaccination for 1-year old						
Unweighted	0.96	0.95	0.01	0.00–0.02	1.01	1.00–1.02
ATE: Average Treatment Effect	0.96	0.95	0.01	0.00–0.02	1.01	1.00–1.02
ATT: Average Treatment Effect for Treated Group	0.96	0.95	0.01	0.00–0.01	1.01	1.00–1.02
ATU: Average Treatment Effect for Untreated Group	0.96	0.95	0.01	0.00–0.02	1.01	1.00–1.02
Complete Vaccination for 2-year old						
Unweighted	0.54	0.48	0.06^a^	0.04–0.09	1.13 ^a^	1.08–1.18
ATE: Average Treatment Effect	0.54	0.49	0.05^a^	0.03–0.07	1.10 ^a^	1.05–1.15
ATT: Average Treatment Effect for Treated Group	0.55	0.50	0.05^a^	0.02–0.07	1.09 ^a^	1.05–1.14
ATU: Average Treatment Effect for Untreated Group	0.53	0.48	0.05^a^	0.03–0.07	1.11 ^a^	1.06–1.16
At Least One Vaccination for 2-year old						
Unweighted	0.97	0.97	0.00	0.00–0.01	1.00	1.00–1.01
ATE: Average Treatment Effect	0.97	0.97	0.00	0.00–0.01	1.00	1.00–1.01
ATT: Average Treatment Effect for Treated Group	0.97	0.97	0.00	0.00–0.01	1.00	1.00–1.01
ATU: Average Treatment Effect for Untreated Group	0.97	0.97	0.00	0.00–0.01	1.00	1.00–1.01

*FF*, Families First program; Risk Difference = predicted probability (in FF – Not in FF); Risk Ratio = predicted probability (in FF/Not in FF)

^a^Statistically significant at alpha = 0.05

Those who were enrolled in the Families First program were significantly more likely to have complete vaccinations than the comparison group at the age of two [ATE RR 1.10 (95 % CI 1.05–1.15), ATT RR 1.09 (95 % CI 1.05–1.14), ATU RR 1.11 (95 % CI 1.06–1.16)]. There were no significant differences between the groups for having at least one vaccination from birth to age two [ATE, ATT, ATU RR 1.00 (95 % CI 1.00–1.01)].

When an interaction term (program x income quintiles) was added to the model for both rural and urban areas, it was not statistically significant. There was no evidence that the program effect differed by income quintile for any of the vaccination outcomes tested.

Gamma sensitivity analyses suggested that our results – (a) one year complete vaccination, (b) one year partial vaccination results, (c) two year complete vaccination, and (d) two year partial vaccination – were each likely robust to unmeasured confounding. For each set of results, there would need to be an unmeasured confounder that both (a) perfectly predicted exposure to the Families First program and (b) accounted for over 50 % of the association between the program and these four sets of results, which is very unlikely.

## Discussion

Overall, this study found an increase in complete vaccination rates at ages 1 and 2 for those enrolled in the Families First program compared to a similar group who were not enrolled. This difference was not observed for the outcome of having at least one vaccination of each immunogen. A comparison of rates by income quintile did not reveal any significant differences.

Several mechanisms can be theorized as to how the Families First program increased vaccination rates, directly and indirectly. The vaccination information contained in the program may have influenced parental behavior directly by increasing the likelihood that they would actively seek out vaccination or well-child care from care providers. Public health nurses eligible to give vaccinations are more easily accessed through the home visitor. In addition, there may have also been an indirect effect through the improvement of the social environment in which families exist, including financial or social resources, which removed barriers to vaccination such as a lack of transportation and childcare, thereby facilitating access to preventive health care. Evidence for home visiting programs improving maternal and child social environments has been assessed by the work of Olds and others with findings of positive effects on a range of social outcomes, such as welfare and food stamp use by mothers [8–11].

The magnitude of the improvement in complete vaccination rates for those enrolled in the Families First program (a range of 6–11 %) is consistent with, albeit slightly lower than, other studies examining vaccination rates for home visiting programs [35]. The modest effect may be explained by program and/or contextual factors. Many parent home visiting programs, including the Families First program, were not originally designed to increase vaccination rates, but rather to address parenting skills and child development [4]. Vaccination content in the Families First curriculum is limited and consists of information intended to educate about the provincial vaccination schedule, as well as to probe regarding the status of childhood vaccinations and well child check-ups. In addition, home visitors in the Families First program are trained laypersons, which may introduce substantial variability in the real world application of the vaccination content of the curriculum and how questions from parents are dealt with regarding vaccinations.

Contextual factors may also explain the magnitude of increase in vaccination rates. The provision of a publicly funded schedule of vaccinations for all Manitobans may reduce the effect of any one intervention at increasing vaccination rates and could potentially have ‘evened’ the playing field between those enrolled in the program and those not in the program. Most families had some exposure to vaccination as evidenced by the relatively high predicted probabilities for both program and non-program groups for having at least one vaccination. This finding also suggests that families in both groups were not philosophically opposed to vaccination, but rather, faced barriers in achieving a complete record of vaccination.

The effect of the Families First program on complete vaccination rates is positive, but may be enhanced with other measures. As discussed previously, home vaccination or home visits promoting vaccination with direct referral to vaccine services could fit well within the scope of current home visiting programs that focus more on cognitive, socio-emotional or child maltreatment outcomes. The large cohort of at-risk families who are directly linked to ongoing public health programming via enrollment in programs such as Families First provides opportunities for incorporating program elements which assess the vaccine status of families and enhance access to vaccination, either during already scheduled home visits or by facilitating referral to specialized vaccination services. This approach may also address cost concerns that have been raised with the high start-up costs of home visiting programs that solely focus on the provision of vaccination. Research is needed to inform policy makers on the best way to integrate these interventions and to further quantify cost-effectiveness in order to successfully improve vaccination coverage rates for children experiencing poverty.

Potential limitations of this study exist. Estimating the true effect of the Families First program on vaccination rates rests on having no residual confounding, which can be difficult to address in observational studies. We have taken this into account in our analysis through the use of propensity scoring; however there is a chance that important variables were omitted. Missing values are another limitation of this study, and while imputing these missing values provided a means of addressing this issue, it is no doubt inferior to having real values. This may have resulted in exposure misclassification. Vaccination outcome misclassification was possible through incomplete or inaccurate documentation in the MIMS database by providers.

Approximately 20 % of births each year in Manitoba did not receive a screen for risk factors. Many of these children were likely from First Nations communities under federal jurisdiction, somewhat limiting the representativeness of the sample. A provincial rather than a federal organization administers the Families First program.

Finally, the effect of the program by income quintile was assessed in the model by using an interaction term, which may not be the optimal approach to assessing this effect. Assessing gaps in program effect for different income quintiles is an area that deserves further research inquiry.

Despite these limitations, there are several strengths of the study. A relatively large and representative sample of at-risk children was analyzed compared to previous studies in this field. Another strength was the ability to link several databases, resulting in a large number of variables that could be used in modeling, thereby reducing the probability of confounding. This was further addressed by the use of propensity scoring. Furthermore, the possibility of omitted variable bias was addressed by employing a Gamma sensitivity analysis. Assessing outcomes for both one and two year olds contributed to the assessment of program effects over time.

## Conclusions

This study reports a moderate increase in complete vaccination rates for one- and two-year old children who were enrolled in the Families First program. The positive effects of the program on complete vaccination rates were seen despite limited program content targeted toward addressing the need for vaccinations. These results suggest a valid method of improving vaccination rates for children experiencing poverty. Program enhancements, such as the addition of home vaccination to program elements, have the potential to increase vaccination rates even further for children enrolled. More research is required to inform policy makers of optimal approaches to improve vaccination rates for children enrolled in home visiting programs.

**Table 6:** Vaccination information in the Families First program curriculum circa 2012

Age group	Questions/Instructions to parents
0–3 months	“What immunizations has she already had?”
	“When is your baby’s next appointment/immunization”
4–6 months	Handout to be given on immunization schedule for Manitoba or to be accessed on Manitoba health website.
10–12 months	“Is he up to date on all of his immunizations?”
	“What has your doctor recommended for the next year as a schedule for routine well baby care and immunizations?”
13–15 months	“Take child for preventive health check-ups and immunizations”
25–30 months	“Take child for preventive health check-ups and immunizations”
	“Are you up-to-date with recommended immunizations?”

## Abbreviations

MIMS: Manitoba Immunization Monitoring System
ATE: Average treatment effect
ATT: Average treatment effect on the treated
ATU: Average treatment effect on the untreated
IPTW: Inverse-probability-of-treatment weights
RR: Risk ratio
